# The plakin family: Potential therapeutic targets for digestive system tumors

**DOI:** 10.1515/jtim-2025-0033

**Published:** 2025-07-30

**Authors:** Changwei Huang, Yixuan Chen, Manoop S. Bhutani, Caixia Wang, Yang Zhou, Jintao Guo, Siyu Sun

**Affiliations:** Department of Gastroenterology, Shengjing Hospital of China Medical University, Shenyang, Liaoning Province, China; Department of Gastroenterology, Hepatology, and Nutrition, The University of Texas MD Anderson Cancer Center, Houston, Texas, USA; Engineering Research Center of Ministry of Education for Minimally Invasive Gastrointestinal Endoscopic Techniques, Shenyang, Liaoning Province, China

**Keywords:** plakin, tumors of the digestive system, cell signaling transduction, clinicopathology

## Abstract

Digestive system tumors remain a global health challenge; however, the mechanisms underlying their tumorigenesis remain unclear. Identifying these mechanisms may facilitate early detection and more effective treatment. Members of the plakin family play crucial roles in cytoskeletal integrity and cell adhesion. Moreover, they regulate key cellular processes implicated in tumor development, including tumor cell migration, proliferation, and signaling. Therefore, exploring the potential roles of the plakin family members in digestive system tumors has attracted increasing attention. In this review, we provide a comprehensive examination of the biological characteristics of the plakin family members and an in-depth analysis of their clinicopathological significance and clinical implications in digestive system tumors. In summary, the plakin family is a translationally valuable diagnostic marker and a potential therapeutic target for digestive system tumors.

## Introduction

The plakin family comprises seven large cytoskeletal crosslinkers.^[1]^ Plakins enable the formation of junctional complexes by linking microfilaments (F-actin), microtubules (MTs), and intermediate filaments (IFs).^[2]^ These cytoskeleton components influence cell polarity, adhesion, migration, and invasion,^[3]^ making plakins crucial for integrating cytoskeletal processes.^[4,5]^

Members of the plakin family have emerged as drivers of various diseases.^[6,7]^ Several studies have focused on their contribution to tumor pathology. However, despite extensive research on plakins in digestive system tumors, a comprehensive review is still lacking. Therefore, in this review, we explore the structures, loci, and functions of plakin family members, as well as their biological, diagnostic, and potential therapeutic roles in digestive system tumors.

## Structure of plakin family members

**Figure 1:**
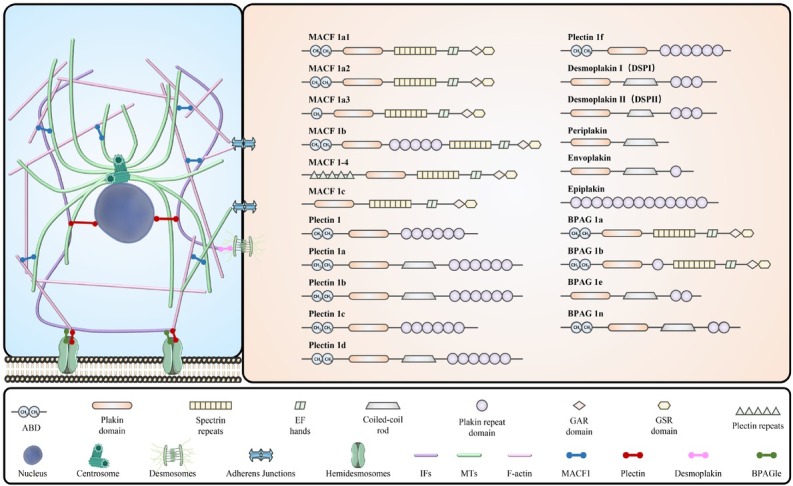
Schematic representation of different members of the plakin family and the roles of selected plakins in cytoskeletal dynamics. ABD: actin-binding domain; BPAG1: bullous pemphigoid antigen 1; CCR: coiled-coil rod; CH: calponin homology; GAR: GAS2-related protein; GSR: glycine-serine-arginine; MACF1: microtubule actin cross-linking factor 1; PRD: Plakin repeat domain; DSP: desmoplakin. Plakin proteins crosslink the three main components of the cytoskeleton: intermediate filaments (IFs), microtubules (MTs), and microfilaments (F-actin). BPAG1e connects IFs to hemidesmosomes; desmoplakin connects IFs to desmosomes; plectin connects IFs to the nucleus; MACF1 binds microfilaments to microtubules.

The mammalian plakin family comprises the following seven members: bullous pemphig oid antigen1 (BPAG1), microtubule actin cross-linking factor 1 (MACF1), plectin, desmoplakin (DSP), envoplakin, periplakin, and epiplakin.^[8]^ Most of these proteins exhibit isoform diversity. Consisting of different unique structural domains that interact with various cytoskeletal components and intercellular junctions, plakins exhibit similar connectivity functions. These structural domains include an actin-binding domain (ABD), a plakin domain, spectrin repeats, helix-loop-helix (EF-hand) motifs, a coiled-coil rod, a plakin repeat domain (PRD), a growth arrest specific 2 (GAS2)-related domain, and glycine-serine-arginine domain (Figure 1).^[9,10]^

The ABD, located at the N-terminus, comprises two calponin homology (CH) domains—namely CH1 and CH^2^—which can bind F-actin and enhance its affinity for actin.^[3,11]^ The plakin domain, composed of α-helices, is present in all plakins except for epiplakin. Plakin domains allow plakins to form connections with various components, such as hemidesmosomes.^[12]^ Spectrin repeats, composed of three α-helices, are only involved in the composition of BPAG1 and MACF1.^[13]^ The PRD, found in various plakins, contains a β-folding and two reverse-helices, which interact with various IFs. Composed of ABD, coiled-coil rod, PRD, and plakin structural domains, plectin stabilizes the cytoskeleton through keratin remapping. Plectin mainly mediates the interaction among three cytoskeletal components, F-actin, MTs, and IFs. BPAG1 isoforms are widely expressed in the digestive system, skin, and reproductive system. Similar to BPAG1, MACF1 isoforms are widely expressed.^[11]^ Although MACF1 and BPAG1 have multiple binding sites, they usually cross-link only one element of the cytoskeleton.^[9]^ In contrast to the other members, DSP, envoplakin, periplakin, and epiplakin, lack the ABD structural domain. DSP, a key component of epidermal cell bridge grains, provides adhesion between cells. Envoplakin and Periplakin, which have similar structures and functions, are found predominantly in the keratinized and non-keratinized stratified squamous epithelia.^[12]^ Interestingly, epiplakin has only one structural domain, the PRD structural domain. It is these unique structural features of the plakin family that determine its function. Plakin proteins regulate the cytoskeletal network, epithelial-mesenchymal transition (EMT), cell adhesion, migration, signal transduction, and other basic biological processes by connecting F-actin, MTs, and IFs.^[14, 15, 16]^

### Pathophysiological role of plakin family members in digestive system tumors

Colorectal cancer (CRC)

**Figure 2:**
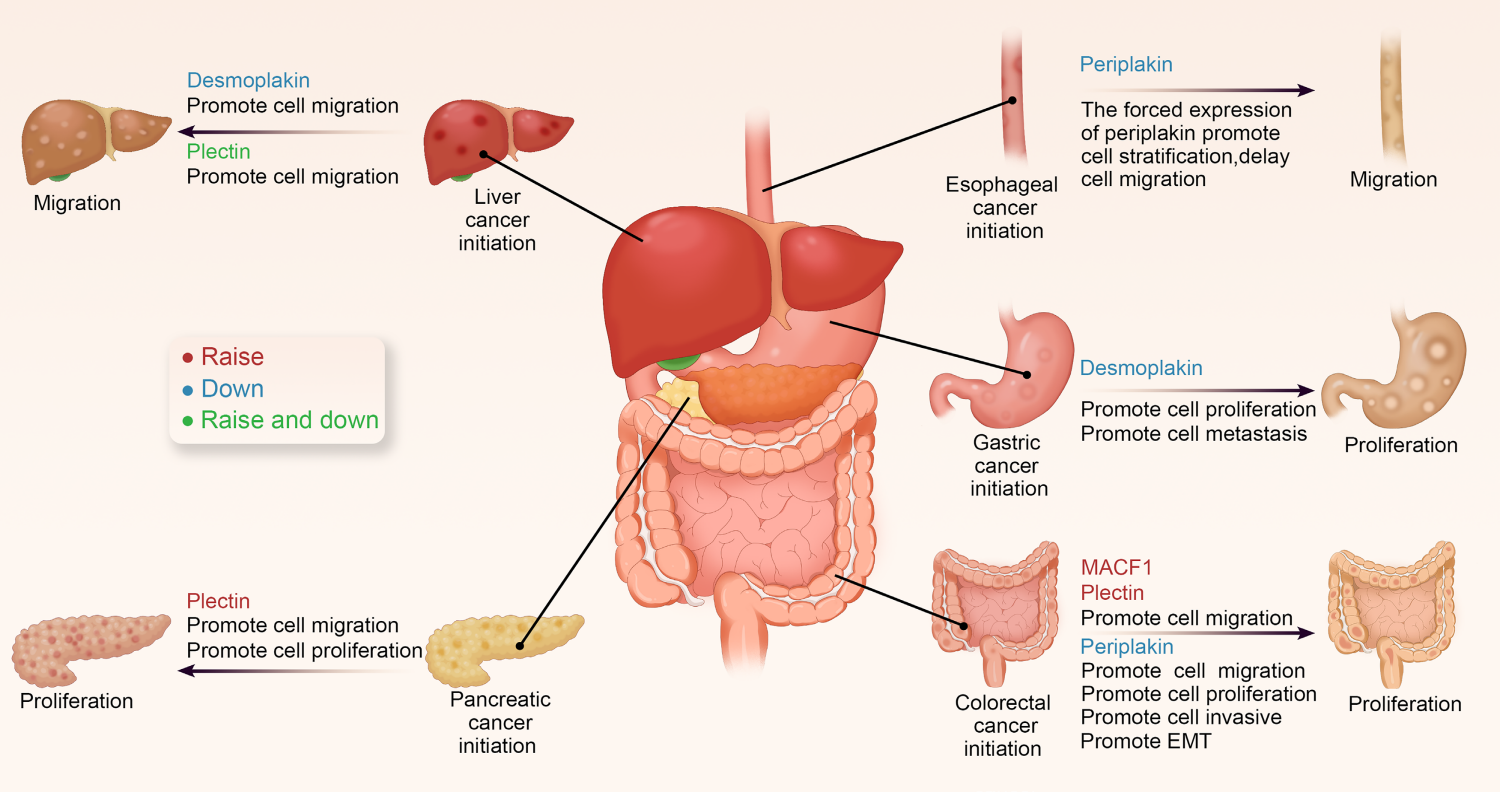
Role of plakins in cancers of the digestive system. Plakins are involved in CRC, PC, liver cancer, and other digestive system cancers. Plakins serve as tumor promoters (indicated in red), tumor suppressors (indicated in blue), or both suppressors and promoters (indicated in green). The major events in solid tumor development are tumor initiation, proliferation, and metastasis. Among these events, cell proliferation, migration, invasion, epithelial-mesenchymal transition (EMT), and apoptosis are either promoted or inhibited. PC: pancreatic cancer; CRC: colorectal cancer.

CRC is one of the most common intestinal tumors.^[17]^ Identifying new key molecules involved in colorectal tumorigenesis is essential for providing novel targets for tumor diagnosis and treatment (Figure 2).^[18,19]^

Previous studies have shown that MACF1 and plectin are upregulated, whereas periplakin is downregulated in human CRC tissues, suggesting that plakins are potential diagnostic biomarkers for CRC. MACF1 is one of the mutationally discordant genes between 19 paired primary and metastatic CRC samples.^[20]^ Compared to classical tumor markers, MACF1 may be a marker for predicting CRC metastasis. The role of plakin in connecting to the cytoskeleton is strongly associated with cell metastasis and migration. Plakin acts primarily as downstream molecules in these processes. For example, lipoprotein receptor-related protein 6 (LRP6) regulates microtubules (MT) assembly in CRC cells *via* MACF1, thereby influencing cytoskeletal remodeling, and its overexpression promotes CRC cell migration.^[21]^ Additionally, plectin can function downstream of transcobalamin 1 (TCN1) in CRC, and its degradation compromises the stability of filamin A and F-actin networks, leading to cytoskeletal damage and CRC cell migration.^[22]^ Furthermore, plectin influences podosomelike adhesion. Knockdown of plectin expression using small interfering ribonucleic acids (siRNAs) impairs the migration, invasion, and adhesion of SW480 cancer cells. Plectin-1k targets podosome-like adhesion and is involved in actin assembly and CRC cell invasion.^[23]^ However, further investigations are required to determine whether it exerts its effects by influencing the cytoskeleton. Notably, MACF1 and plectin act as tumor promoters in CRC by affecting the cytoskeleton and intercellular interactions, thereby promoting cell migration. While periplakin, as a tumor suppressor in CRC, not only affects cell migration but also plays a role in cell proliferation and invasion and EMT.^[24]^ In summary, these findings highlight the significant role of plakin family members in CRC tumorigenesis, suggesting their potential as diagnostic biomarkers and therapeutic targets. Understanding how plakins contribute to the dynamic interactions between the cytoskeleton and cellular signaling pathways may provide new avenues for targeted treatments and early detection strategies in CRC.

#### Pancreatic cancer (PC)

PC is one of the most aggressive and incurable malignancies.^[25, 26, 27]^ Over the past decades, DSP and plectin have been considered potential diagnostic targets for PC. DSP, an epithelial tissue marker of PC, is upregulated during EMT, while epithelial tissue markers are downregulated during PC progression. However, previous studies have only established an association between DSP and PC, with the detailed mechanisms yet to be fully elucidated.^[28,29]^ In contrast to DSP as a marker in EMT, plectin has been shown to play an active role in cell migration and growth. Babicky *et al*. were the first to demonstrate the role of plectin in PC cell migration. Exposed to the ligand macrophage-stimulating protein, recepteur d′origine nantais (RON) is transferred from the paranuclear cytoplasm to the cell surface. The RON receptor activation in PC cells leads to its binding with plectin and integrin beta 4, major components of hemidesmosomes that anchor cells to the extracellular matrix and inhibit cell migration.^[30]^ These findings suggest that plectin negatively regulates cell motility. Studies on exosomes have demonstrated that plectin serves as an intracellular scaffolding protein under normal physiological conditions. However, plectin is upregulated in PC cells, and its abnormal upregulation results in its localization to secreted exosomes, which may promote PC growth.^[31]^ Knocking down plectin using short hairpin RNAs (shRNAs) reduces exosomal plectin secretion, consequently inhibiting PC cell proliferation, migration, and invasion.^[31]^ Further studies are needed to clarify their exact roles in disease progression and validate their potential for clinical use.

#### Liver cancer

Liver cancer is the seventh most common cancer and second most common cause of cancer-related deaths.^[32]^ Hepatocellular carcinoma (HCC) is the most common type of liver cancer.^[33]^ Members of the plakin family play a role in HCC cell migration. DSPs are primarily involved in EMT and serve as epithelial tissue markers. In contrast, DSP knockdown in Hep3B and HepG2 cells using siRNAs increased cell migration, not by triggering EMT but due to the absence of DSPs.^[34]^ Controversial findings regarding the role of plectin in HCC cell migration have shown that plectin knockdown in human hepatocytes activates adherent spot kinase and ras-related C3 botulinum toxin substrate -Guanosine-Triphosphate hydrolase (Rac1-GTPase) to promote cell migration.^[35]^ These findings align with those of HCC cytopathology studies showing low plectin expression in HCC cells.^[36]^ Furthermore, invasion assays have demonstrated a positive correlation between low plectin levels and high single-cell migration capacity, although no significant differences were observed.^[36]^ In contrast to previous findings, plectin expression and migration assays in four cell lines (MHCC97L, MHCC97H, human hepatocarcinoma cells [HCCLM3], and Human hepatocellular carcinomas [HPG2]) revealed that plectin was significantly upregulated in HCC tissues and promoted HCC cell migration, whereas plectin knockdown using shRNAs inhibited HCC cell migration. A potential mechanism is EMT inhibition *via* the extracellular signal-regulated kinase 1/2 (ERK1/2) signaling pathway.^[37]^ Moreover, plectin is associated with individual cell migration and may be closely related to collective cell migration. It may interact with Netrin-1 to enhance overall HCC cell migration.^[38]^ In HCC, increased extracellular matrix stiffness upregulates plectin expression, increasing F-actin polymerization to promote cell migration.^[39]^ Although both DSP and plectin can affect cell migration, plectin has been shown to be potentially associated with the collective migration of cells. Furthermore, in liver cancer stem cells, neuronal cell adhesion molecule （NRCAM） mediates β-catenin signaling to activate EMT *via* MACF1.^[40]^ Further investigations are warranted to explore its potential as a therapeutic target for inhibiting HCC cell migration and EMT.

#### Other digestive system tumors

The plakin family has also been investigated in other digestive system tumors, including gastric and esophageal cancers. Whole-exome sequencing (WES) of gastric cancer samples revealed that MACF1 mutations are moderately common in patients with peritoneal metastases.^[41,42]^ A study assessing circulating tumor cell-free deoxyribonucleic acid (DNA) (cfDNA) from patients with gastric cancer found a higher MACF1 mutation in stage IV than in stages I-III, suggesting an association between MACF1 expression and gastric cancer metastasis. However, the underlying mechanisms remain unclear.

DSP promotes gastric cancer growth and metastasis. The establishment of the knockdown and overexpression models of DSP in gastric cancer have revealed that its overexpression inhibits gastric cancer cell proliferation and promotes apoptosis, whereas its knockdown showed the opposite effects. Moreover, Transwell assays revealed that DSP knockdown enhanced the invasion and migration of gastric cancer cells.^[43]^

Proteomic studies have revealed that periplakin is aberrantly expressed in esophageal cancer and may be closely associated with lymphatic metastasis.^[44]^ Furthermore, periplakin overexpression has been shown to promote cell lamination, facilitate cell and extracellular matrix adhesion, and retard cell migration, consistent with the trend of low periplakin expression observed in esophageal cancer.^[45]^

Both DSP and periplakin play active roles in different digestive tumors,^[24,33,43,45]^ although their mechanisms of action differ. A deeper understanding of cytoskeletal junction proteins is required to explore how they affect cellular mechanotransduction and the tumor microenvironment, thereby influencing different digestive tumors.^[46,47]^

#### Shared and member-specific effects

**Figure 3:**
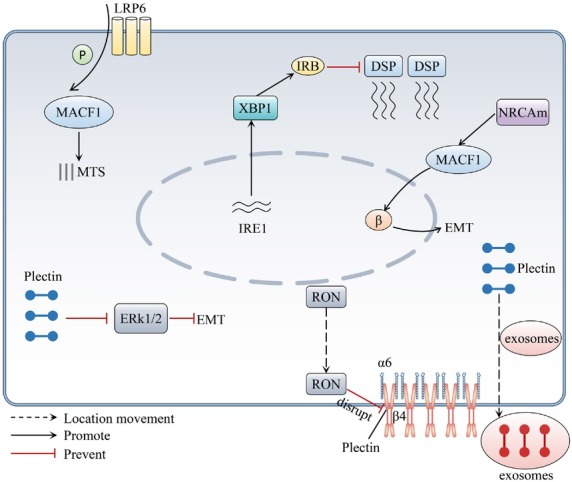
Schematic representation of how the plakin family affects cell migration and EMT of digestive tumor cells. The plakin family is involved in the cytoskeleton and intercellular interconnections and affects tumor cell migration and EMT through signal transduction pathways. The activation and inhibition of different proteins are indicated by solid arrows and solid double lines, respectively. Dashed arrows indicate the translocation of proteins. P: phosphorylation; β: β-catenin; DSP: Desmoplakin.

In various digestive system tumor cells, plakin family members are closely associated with cell migration, despite their up- and down-regulation. The specific mechanisms of action vary. However, they are associated with altered cytoskeletal networks and intercellular connectivity. Additionally, they affect signaling pathways, including the wnt/β-catenin signaling pathway (Figure 3).^[21,22,30]^ Additionally, the expression of most of the members can be regulated by RNA and further affect migration.^[23,31]^

Current research suggests that plectin promotes cell migration in HCC, PC and CRC. Interestingly, the promotion of migration by plectin in HCC may be related to the collective migration of cells.^[38]^ Compared to other members of the plakin family, plectin plays a dual role in digestive system tumors, exerting both pro- and anticancer effects. Similarly, integrin α6β4 can play a dual role in tumors, recruiting plectin to the plasma membrane. We speculate whether the dual action of plectin stems from the mislocalization of pletin recruited to the plasma membrane by integrin α6β4. That warrants further experimental verification.^[48]^ In gastric and liver cancers, DSP affects cell migration; however, in PC, current studies only show that it acts as an epithelial marker for EMT.^[34,43]^ Further investigation is needed to determine the effect of DSP on PC cell migration. Envoplakin and Periplakin possess similar structures and functions. Currently, it has been shown that periplakin affects cell migration, cell proliferation, and EMT in esophageal and CRC.^[24,44]^ Understanding the function of envoplakin, particularly its impact on cell migration, is crucial. Although similar structures exist between plakin families, between individual digestive system tumor cells. However, their structures are not identical, which may potentially explain the differences in function.

### Potential clinical applications of plakin family members in digestive system tumors

Digestive system tumors remain a significant cause of tumor-related deaths worldwide, highlighting the importance of identifying biomarkers for early detection and translating the findings into clinical practice.^[49,50]^ Recent studies have identified members of the plakin family as potential biomarkers for the diagnosis and prognosis of digestive system tumors (Table 1).^[51-53]^

**Table 1:** Potential clinical applications of plakin family members as biomarkers for digestive system tumors

Cancer type	Study object	Study subjects and control sample size	Test samples	Test method (s)	Potential clinical application	Ref.
PDAC	PDAC, chronic pancreatitis, and normal pancreata samples	4 normal pancreata, 15 chronic pancreatitis, 14 PanIN I, 26 PanIN II, 15 PanIN III, 41 PDAC, 8 liver metastases, 11 lymph node metastasis, 10 matching primary tumors, and 9 peritoneal metastasis samples	PDAC, pancreatitis, and normal pancreata samples	IHC and western blot analysis	Diagnostic, prognostic, and metastatic biomarkers	[53]
PDAC	PANC-1, MIA PaCa-2, HPAC, Mpanc-96, and BXPC-3 cells			*In vivo* bioluminescence imaging, luciferase assays, and immunohistochemistry	AAV-plectin 1-targeting peptides preferentially targeted PDAC cell lines.	[68]
PDAC	Panc-1 and LO2 cells			MRI and *in vitro* laser- scanning confocal microscopy	Plectin-targeting iron oxide nanoparticles used as imaging-contrast agents for early PDAC diagnosis	[62]
PDAC	MIA Paca-2 cells			Cellular fluorescence-based microscopy images	Plectin-targeting peptide attached to magnetofluorescent nanoparticles helped to detect PDAC cells.	[60]
PDAC	PANC-1 and MIA-Paca2 cells			Multiphoton microscope	Plectin-targeted lipid microbubbles help detect PDAC cells.	[65]
PDAC	Pancreatic mass in patients	85 patients with pancreatic masses	Pancreatic mass samples were obtained by EUS-FNA.	Cytology, KRAS mutations, plectin staining, and final diagnosis	Plectin-1 staining can help improve the diagnostic accuracy of EUS-FNA.	[57]
PDAC	ASPC-1 and PANC-1 cells			*In vitro* cell-proliferation assay, *in vivo* tumor-growth inhibition, and hematoxylin and eosin staining	Plectin-1-targeted multifaceted peptide-assisted one-pot synthesis of gold nanoparticles can help gemcitabine delivery.	[71]
PDAC	MIA-Paca-2 and XPA-1 cells. MIN6 Mice			MRI, *in vivo* optical imaging, and hematoxylin and eosin staining	Plectin-1-targeted fluorescence and MR dual-functional nanoparticles can be used to visualize PDAC.	[61]
PDAC	PANC 1 and hTERT-HPNE cells			Multiphoton imaging	Plectin-targeted lipid microbubbles and multiphoton imaging can help detect PDAC.	[63]
PDAC	ASPC-1, Capan2 L929, PANC-1, T3M4, BXPC3 HPDE6-C7, SW1990, HUVEC cells, and MiceL929			*In vivo* confocal fluorescence laser microscopy of bipeptides, *in vivo* optical and MRI of Gd-Cy7-PTP/RGD, and optical imaging-guided surgery of Gd-Cy7-PTP/RGD	Plectin/integrin-targeted bispecific molecular probe for MRI/NIR imaging of PDAC	[64]
PDAC	Panc-1 cells			Cell viability assays	Plectin-targeted chaperonin-GroEL can promote PDAC cell apoptosis.	[70]
PDAC	CPI-613 and LY2109761			*In vivo* antitumor activity detected with Picro Sirius Red staining and IHC staining of α-SMA	Plectin-targeted, tumor- responsive nanopolyplex targeted PDAC cells and stroma.	[69]
PDAC	Patients with PC	32 patients with PC	CTCs	CTC platform and a C-microfabricated porous filter	Plectin can help identify CTCs during early-stage PDAC.	[58]
CRC	Colorectal adenocarcinoma and tubular adenoma	25 patients with colorectal adenocarcinoma and 10 patients with tubular adenoma	Cancer samples	IHC	Plectin may serve as an oncofetal biomarker.	[49]
CRC	Patients with CRC, healthy control subjects, and fetuses	30 cancer samples, 30 control samples, and 30 fetus samples	Serum samples	2D DIGE coupled with a Finnigan LTQ-based proteomics approach. GC-MS instrument integrated with a commercial mass spectrometry library	MACF1 may serve as an oncofetal biomarker.	[50]
CRC	CT 26 colon tumor model			Subcutaneous implanted tumor model	Plecstatin can target plectin and inhibit colorectal tumors.	[51]
CRC	HCT116, HT-19, and HCT-115 cells			Colony-formation assay, flow cytometry analysis of apoptosis, and spheroid growth assays	Plectin-1 treatment reduced spheroid growth and decreased the colony-forming ability of colon adenocarcinoma cells.	[52]
Esophageal cancer	Primary esophageal squamous cell carcinoma samples and non- tumor samples	12 tumor samples and 12 non-tumor samples	Tumor cell proteins and non-tumor cell proteins	2D DIGE, immunoblotting, and IHC	Periplakin is a potential marker for detecting early esophageal cancer and evaluating tumor progression.	[41]
Esophageal cancer	Tumor cells and adjacent normal mucosal cells	72 paired tumor cell and mucosal cell samples	Cancer cells protein	2D-PAGE, MS-based protein identification, and western blot analysis	Periplakin may be used to diagnose esophageal cancer.	[75]
Gastric cancer	Gastric carcinoma samples from patients	74 samples, including intestinal, diffuse, mixed, and indeterminate adenocarcinomas	Cancer tissue sample	WES	MACF1 may serve as a marker of metastasis in gastric cancer.	[38]
Gastric cancer	Stage IV gastric cancer samples	56 stage IV gastric cancer samples	Cancer tissue sample	Next-generation sequencing	MACF1 expression may serve as a potential biomarker for stage IV gastric cancer.	[73]
Liver cancer	Human plasma and liver tissues from patients with liver cancer	3 patient samples	Plasma and cancer tissues	Targeted phosphopeptide analysis and immunoblotting	Plectin may serve as a potential phosphobiomarker in liver cancer.	[74]

2D DIGE: two-dimensional difference gel electrophoresis; 2D-PAGE: two-dimensional polyacrylamide gel electrophoresis; AAV: adenoassociated virus; CTC: circulating tumor cell; EUS-FNA: endoscopic ultrasound-guided fine-needle aspiration; GC: gas chromatography; IHC: immunohistochemistry; LTQ: linear ion trap mass spectrometer; MRI, magnetic resonance imaging; MS: mass spectrometry; PanIN: pancreatic intraepithelial neoplasia; PDAC: pancreatic ductal adenocarcinoma; WES: whole-exome sequencing; NIR: near-infrared; α-SMA: α-smooth muscle actin; MS-based protein: mass spectrometry-based protein; Ref.: references.

#### CRC

Immunohistochemical analyses of colorectal and tubular adenomas revealed that plectin expression was substantially higher in tumor cells than in normal colorectal mucosal cells, indicating its potential as an early diagnostic biomarker for early-stage CRC and precancerous lesions.^[54]^ Additionally, phosphorylation of LRP6, which functions upstream of MACF1,^[55]^ was strongly associated with the tumor-node-metastasis stage, Dukes stage, and poor prognosis, suggesting that MACF1 is related to the prognosis of CRC.^[21]^ Interestingly, a clinical study evaluating programmed death-ligand 1 （PD-L1） inhibitors combined with radiation for treating advanced colorectal cancer demonstrated that MACF1 expression was upregulated in treatment-responsive patients, suggesting that MACF1 serves as a potential marker for immunotherapy response.^[56]^ Nevertheless, more clinical studies are required to validate these findings.

Plecstatin-1 is an organometallic chemotherapeutic agent targeting plectin. It inhibits tumorsphere growth and induces changes in its morphology and structure by targeting plectin to affect the cytoskeleton. It also blocks tumor growth and induces G0/G1 cell cycle arrest in colon cancer cell lines, which reduces mitochondrial membrane potential, reactive oxygen species levels, and tumor cell proliferation. In a mouse model of colorectal cancer, it not only showed good tolerance but also resulted in a significant reduction in tumor volume.^[57,58]^ However, the translational applicability of these treatment modalities requires further investigation.

#### PC

Immunohistochemistry of normal pancreatic, chronic pancreatitis, and PC tissues indicates that normal pancreatic and pancreatic tissues are negative for plectin, whereas PC tissues are 100% positive. Additionally, plectin expression increases during PC development and can be used to distinguish early pancreatic intraepithelial neoplasia (PanIN) I and II lesions from PanIN III and pancreatic ductal adenocarcinoma.^[59]^ Plectin is closely related to tumor staging and metastasis, making it a strong prognostic marker.^[59]^ Plectin is more specific for invasive and preinvasive PC than classical markers of PC, including carbohydrate antigen199 (CA199) and carcinoembryonic antigen (CEA).^[60, 61, 62]^ A study analyzing 85 patients with pancreatic masses sampled using endoscopic ultrasound-guided fine-needle (EUS-FNA) aspiration, an essential method for early diagnosis of PC, showed that the sensitivity, specificity, and accuracy of the samples for histological diagnosis were 81%, 80%, and 79%, respectively.^[63]^ Kirsten ratsarcoma viral oncogene homolog (KRAS) mutation assessment, in combination with histological examinations, showed sensitivity, specificity, and accuracy of 93%, 87%, and 92%, respectively. Furthermore, the combination of histological assessments, KRAS mutation analysis, and plectin staining increased the diagnostic sensitivity, specificity, and accuracy to 96%, 93%, and 95%, respectively. These findings indicate that plectin staining can improve the diagnostic ability of endoscopic ultrasound-guided fine-needle aspiration (EUS-FNA)-acquired pancreatic tumor samples.^[63]^ However, the diagnostic efficacy of combining plectin with P53, another common mutation site in PC, remains unclear. Researchers have identified plectin as a biomarker for circulating tumor cells in portal and peripheral blood samples and found plectin-positive circulating tumor cells in 43.8% and 50% of portal and peripheral blood samples, respectively. However, no plectin-positive circulating tumor cells were detected in samples from healthy individuals.^[64]^ Nevertheless, its comparison with immunofluorescence-fluorescence *in situ* hybridization remains to be explored.^[65]^ These findings suggest that plectin is a reliable marker for the diagnosis of PC; however, large multicenter randomized controlled studies are required to confirm these findings.

Plectin can be used as a targeting agent for PC. A study using a mouse model of PC revealed that a plectintargeted peptide conjugated to magnetofluorescent nanoparticles enabled specific detection of PC cells in normal pancreatic tissue using confocal microscopy with live tissues. However, further studies are required to elucidate the clinical implications of these findings.^[66]^ Additionally, plectin-targeting peptides and plectin antibodies can enhance the accumulation of nanoparticles, microbubbles, 1,4,7,10-tetraazacyclododecane-1,4,7,10-tetraacetic acid-based fluorescent probes, and imaging probes in pancreatic tumor cells, facilitating PC detection.^[67, 68, 69, 70, 71]^ Magnetic resonance imaging and confocal microscopy used to detect plectin antibody-conjugated, targeting iron oxide nanoparticles in PC and normal pancreatic cell lines revealed high plectin expression in PC cell lines but not in normal pancreatic cells.^[67,68]^ Previous *in vitro* studies have shown that lipid microbubbles targeting plectin bind specifically to PC cell lines, enabling rapid detection of the absence of PC cells on the surfaces of cut tissues after PC surgery.^[68,71]^ Furthermore, a bimolecular probe based on plectin and integrin showed good diagnostic results for PC tissues both *in vitro* and *in vivo*.^[64]^ Moreover, a novel DOTA-based plectin-targeted molecular probe has also shown promise for the diagnosis of PC.^[72]^ A study targeting plectin in three patients with pancreatic ductal adenocarcinoma reported no adverse events, demonstrating its safety.^[73]^ Further studies are needed to validate their clinical performance and safety.

Plectin is also a potential target for drug delivery. Researchers have developed various delivery routes, including polymeric nanoparticles, gold nanoparticles, targeted adeno-associated virus particles, and natural-protein drug-delivery systems that can target cancer-specific plectin and promote targeted delivery to PC cells.^[74, 75, 76, 77, 78]^ Despite *in vivo* and *in vitro* validation of these delivery modalities, these modes of administration are yet to be implemented clinically, warranting further investigations to determine their therapeutic efficacy and safety.

#### Other digestive system tumors

Proteomic analyses have shown aberrant expression of periplakin in esophageal cancer.^[44]^ Immunohistochemical staining studies have demonstrated that periplakin is predominantly localized at the cell border in normal esophageal tissues. However, in atypical hyperplastic tissues, it shifts to the cytoplasm during early-stage cancers, where it is barely expressed. This suggests that periplakin may be useful as a biomarker for diagnosing early esophageal cancer.^[79]^ Studies have also indicated a strong association between MACF1 with gastric cancer stage and metastasis, suggesting it as a potential prognostic biomarker. In a study, WES analysis of 74 gastric cancer samples revealed a high rate of MACF1 mutations in patients with gastric cancer who had peritoneal metastases.^[41]^ Additionally, next-generation sequencing of cfDNA from 56 patients with stage IV gastric cancer showed that MACF1 was the most frequently mutated gene in patients with metastases and cfDNA.^[80]^ Furthermore, phosphorylation proteomics analyses revealed plectin as a potential phosphate biomarker for HCC.^[81]^ Immunohistochemical staining of 18 HCC and normal tissue samples showed that plectin was significantly downregulated in HCC samples,^[82]^ suggesting its potential as an HCC diagnostic marker. Overall, these studies indicate that the plakin family holds promise as potential biomarkers for various digestive system tumors. Nevertheless, further clinical studies are necessary to validate their diagnostic performance and clinical applicability. Additionally, plecstatin-1 (plectin inhibitor) was well tolerated and effective in inhibiting HCC progression in a mouse HCC model.^[83]^

#### Shared clinical applications and member-specific potential

Immunohistochemistry, WES, and proteomics studies using clinical samples have demonstrated that members of the plakin family can be used as potential diagnostic markers for digestive system tumors.^[41,44,59,79]^ Immunohistochemistry results have shown that plectin has better diagnostic results for PC because of higher sensitivity and specificity. However, limited research exists on prognostic markers, with relevant studies currently focusing on the effects of pletin on PC and MACF1 on CRC.^[54,59]^ Current translational research on plectin as a marker for PC is more advanced, exploring not only the potential advantages of plectin over traditional markers but also its use as a targeting agent to improve diagnostic yield in combination with advanced endoscopic techniques such as endoscopic ultrasound (EUS).^[60,63]^ In contrast, the role of other plakin family members for other digestive tumors (especially early tumors) should be further explored. Current research on treatment focuses on the targeted delivery of plectin for PC and the use of plectin-targeting agents for CRC and HCC.^[57,74]^ Targeting agents against other family members of plakin, along with drug development, is highly desirable. Additionally, whether the plakin family is involved in tumor drug resistance requires further investigation. It is noteworthy that most translational studies have focused on plectin compared to other family members. This may be related to its abnormal localization to the cell membrane.

## Conclusions

The plakin protein family plays a role in cytoskeletal dynamics, cell migration, proliferation, and other biological processes. Abnormal plakin expression is strongly associated with the development of digestive system tumors. Although high-quality clinical studies are required to confirm this finding, plakins present potential diagnostic and therapeutic targets for these cancers.

**Figure 4:**
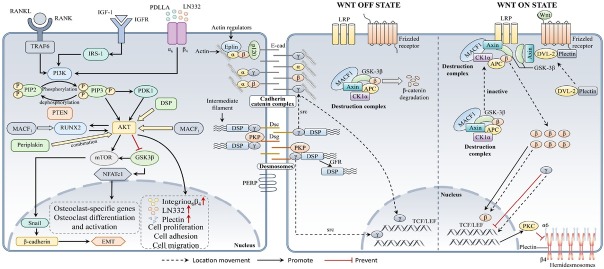
Schematic diagram showing how members of the plakin family affect the phosphatidylinositol 3-kinase (PI3K) -protein kinase B (AKT) and Wnt signal-transduction pathways. Several members of the plakin family can act on specific links in this pathway, thereby affecting the activity of the pathway. The activation and inhibition of different proteins are indicated by solid arrows and solid double lines, respectively. Dashed arrows indicate the translocation of proteins. α: α-catenin; β: β-catenin; γ: γ-catenin; p120: p120-catein; PKP: plakophilin; src: Src-family tyrosine kinases.

Due to similar structural domains, the plakin family can play a role in cytoskeletal connections, cellular interconnections, and signal transduction pathways in digestive tumor cells. In other cells, plakin family members were shown to be closely related to the Wnt-β-catenin and phosphatidylinositol 3-kinase-protein kinase B pathways (Figure 4). A deeper understanding of these mechanisms will help us further explore how the plakin family functions in digestive system tumor cells.^[43,84, 85, 86, 87, 88, 89, 90, 91, 92, 93]^

Individual plakin family members exhibit varying effects on tumor progression. Some proteins promote tumorigenesis, while others have opposing roles.^[32,34]^ This discrepancy may result from the structural differences in various tumors. Additionally, the distinct cytoskeletal junction proteins connecting different components could be a contributing factor. For example, in contrast to the other members, Epiplakin consists only of plakin repeat domains (PRDs) that bind only to intermediate filament (IF). This may affect tumor cells differently. Nevertheless, further studies employing advanced technologies, such as omics, gene knockout, and knock-in models, may help elucidate these underlying reasons.^[94,95]^ Moreover, while most studies have focused on tumor cells, exploring interactions between plakin family proteins and stromal or immune cells facilitated by single-cell sequencing and immune microenvironment research^[96,97]^ will provide additional insights.^[98]^ Additionally, factors such as aging and circadian rhythms have been shown to be closely associated with digestive tumors, making it urgent to explore the role played by the plakin family.^[99,100]^

Current research suggests that the plakin family members hold promise as diagnostic and prognostic biomarkers for digestive system tumors.^[101]^ Particularly, their diagnostic performance offers unique advantages compared to other classical biomarkers. However, large-scale cohort studies are essential for validating these findings before clinical application. Currently, plectin is being used as a targeted contrast agent for PC and although various plectin-targeting molecules have shown promise in preclinical single-center studies, more multicenter trials are needed to compare their effectiveness. Based on these studies, expanding the use of plakin family members as targeted contrast agents for other digestive system tumors is highly desirable. Additionally, combining the plakin family with clinical techniques such as liquid biopsy and needle-based confocal micro-endoscopy could improve the early diagnosis of digestive system tumors.^[102,103]^ However, multi-center prospective studies are needed to determine the most accurate diagnostic combination. Although prognostic studies have shown that the expression of plakin family members correlates with the stage of digestive tumors, no cohort studies have demonstrated an association with survival outcomes in patients with digestive tumors. Interestingly, plectin demonstrates strong efficacy as a diagnostic target and in targeted drug delivery for PC compared to other plakin family members for other digestive tumors. This outcome may be because plectin can exist as an exosome that is more readily bound between cells.^[32]^ Furthermore, the development and combination of plakin family inhibitors with other anticancer drugs may provide effective antitumor therapies. Except for plectin, no clinical studies currently focus on inhibitors targeting the plakin family.^[104,105]^ Some researchers have developed an agonist, I-3, which targets periplakin and has shown better results in treating vitiligo in *in vivo* animal tests.^[106]^ A monoclonal antibody manifestation targeting plectin mislocalized on the cell surface of ovarian cancer cells showed promising results in *in vivo* and *in vitro* assays.^[107]^ While this holds promise, challenges remain in developing agonists and inhibitors of plakin family members in digestive tumors. No studies have shown the presence of enzyme- and receptor-binding sites for the plakin protein family in digestive system tumors, and this may lead to difficulties in developing agonists and inhibitors due to the lack of binding sites. Except for plectin, most cytoskeletal junction proteins are usually present in the cytoplasm. These proteins are developed for interaction with agonists and inhibitors, which are required to have good lipid solubility and suitable molecular weight to pass through the cell membrane. Additionally, because of their widespread role in the cytoskeleton, their agonists and inhibitors may disrupt the cytoskeleton of normal cells, potentially resulting in toxicity and adverse effects.

Translational research exploring the plakin family in digestive system tumors is imminent. A summary of current clinical studies (Clinicaltrials, Trial search.who. int) is warranted. Currently, there are phase I/II clinical trials of plectin-targeting drugs in solid tumors, including PC (NCT05074472). More interestingly, there are also ongoing studies exploring the diagnostic role of plectin in cholangiocarcinoma obtained by ERCP (Endoscopic Retrograde Cholangiopancreatography) (NCT06651346). EUS and ERCP are promising diagnostic and therapeutic techniques for the biliopancreatic system. Given the potential of the plakin family, especially plectin in PC, more future clinical studies in this direction are warranted.

As research into the mysteries of the role of the members of plakin family in digestive system tumors progresses, a more comprehensive understanding of their function will help pave the way for novel diagnostic and therapeutic strategies for future targeted interventions.
